# Utilizing TabNet Deep Learning for Elephant Flow Detection by Analyzing Information in First Packet Headers

**DOI:** 10.3390/e26070537

**Published:** 2024-06-22

**Authors:** Bartosz Kądziołka, Piotr Jurkiewicz, Robert Wójcik, Jerzy Domżał

**Affiliations:** Institute of Telecommunications, AGH University of Krakow, 30-054 Krakow, Poland

**Keywords:** flows, flow table, elephant, mice, traffic engineering, machine learning, TabNet, feature importance, input information

## Abstract

Rapid and precise detection of significant data streams within a network is crucial for efficient traffic management. This study leverages the TabNet deep learning architecture to identify large-scale flows, known as elephant flows, by analyzing the information in the 5-tuple fields of the initial packet header. The results demonstrate that employing a TabNet model can accurately identify elephant flows right at the start of the flow and makes it possible to reduce the number of flow table entries by up to 20 times while still effectively managing 80% of the network traffic through individual flow entries. The model was trained and tested on a comprehensive dataset from a campus network, demonstrating its robustness and potential applicability to varied network environments.

## 1. Introduction

The term *elephant flows* refers to the most substantial data transfers across the Internet, which, despite their limited numbers, usually carry the majority of the traffic. In contrast, the numerous *mouse flows* constitute a large portion of the flow count but account for only a minor fraction of total traffic. This imbalance surpasses the conventional 80/20 ratio defined by the Pareto principle. Recent studies, including references such as [1,2], have revealed that a mere 0.2–0.4% of all flows might be responsible for as much as 80% of the entirety of Internet traffic, showcasing an extreme concentration of data within a small fraction of flows.

The approach of flow-based traffic engineering has recently emerged as a powerful technique for addressing the challenges of escalating network demands while preserving the quality of service (QoS) [3,4,5]. This strategy involves assigning a unique forwarding entry to each flow in the switch’s memory, with each entry detailing the subsequent hop along the flow’s path. This arrangement allows for the use of varied paths for flows that share the same source and destination, thereby facilitating multipath routing. Moreover, paths for incoming flows can be chosen based on present or expected network congestion, enabling an adaptive routing that effectively avoids congested links. Furthermore, this method of flow-based adaptive routing is known to offer higher stability compared to conventional dynamic load-balancing techniques.

The primary issue in flow-based traffic engineering arises from the fact that the number of concurrent flows in a network often exceeds the capacity of the flow tables within switches [6]. Furthermore, in centralized software-defined networks (SDNs), there is a bottleneck concerning the controller’s ability to handle new flow setups due to its throughput limitations. Beyond the issue of capacity, having fewer entries in the flow tables can lead to faster table lookups, thereby enhancing the packet switching speed. One viable approach to mitigate these challenges involves dedicating entries exclusively to the largest flows. Consequently, the majority of smaller flows could be directed along default, shortest-path routes. This strategy significantly reduces the number of flow table entries while still effectively managing a substantial volume of traffic through specialized, flow-specific entries. It is crucial to predict these values as early as possible to quickly establish individual entries and route most of their packets through specific paths. Ideally, flows should be classified with their initial packet to prevent mid-connection rerouting, which can disrupt transport protocols’ path state estimations. Additionally, we find that first packet classification is mostly an unexplored field, especially within the context of SDN traffic engineering. Therefore, we decided to focus solely on the first packet classification to fill this gap.

Our research evaluates the efficacy of the TabNet [7] deep tabular data learning architecture, with a particular focus on metrics vital for traffic engineering within SDNs. The key contributions of our work are as follows:

- **Traffic Coverage:** We examine the volume of traffic managed by flows classified as elephants following their identification, termed *traffic coverage*.
- **Flow Table Reduction:** We analyze the reduction in the necessity for individual flow entries in the tables, denoted as *flow table operation reduction*.
- **Entropy Analysis:** We provide an analysis of the average information entropy contained in each 5-tuple field in packet headers.
- **Feature Significance:** We identify which 5-tuple fields were the most significant for predictions.

## 2. Related Work

The strategy of selectively managing elephant flows dates back to 1999 when the idea of adaptively routing substantial data flows was introduced [8]. Initially, due to the hardware constraints of the era, the concept was largely theoretical and confined to academic discussions. However, the rise of SDNs has revitalized interest in this approach. In the contemporary networking landscape, a controller with comprehensive insight into network dynamics is well positioned to efficiently oversee large flows, leveraging the advanced capabilities of SDNs.

The Hedera traffic engineering system, unveiled in [9], was created to dynamically reroute flows through an embedded controller once they surpassed a predefined threshold, guiding these flows along paths selected in real time. It presupposes that edge devices are responsible for collecting comprehensive flow statistics via OpenFlow counters, with a focus on optimizing the performance of non-edge devices. DevoFlow, introduced in [10], emphasizes the management of elephant flows by implementing sampling techniques and utilizing threshold values for their identification. Nevertheless, the evaluation of DevoFlow’s effectiveness is conducted based on the network’s aggregated performance, not on flow table characteristics. A similar approach to DevoFlow is explored in [11], where elephant flows are identified at edge devices using an adapted Bloom filter. This method’s underlying traffic model, assuming a disproportionate contribution of 20% of the flows to 80% of the traffic, diverges significantly from real-world data distributions, as recent research, such as [1,2], suggests a much more tail-skewed distribution.

The referenced studies primarily employ rudimentary techniques such as sampling, counters, and threshold settings for the detection of large flows. However, there has been a shift towards more sophisticated, machine-learning-based approaches in recent years. For instance, a decision tree model dedicated to identifying elephant flows was introduced and assessed in [12], with a particular emphasis on the *accuracy* of detection. In another study, ref. [13] by Poupart et al. explored the capabilities of three different machine learning (ML) strategies for estimating flow sizes and categorizing them as elephant flows. Their analysis was based on a comprehensive dataset of three million flows, covering both TCP (Transmission Control Protocol) and UDP (User Datagram Protocol). Their evaluation focused on two principal metrics: the success rate in correctly identifying large flows (*true positive rate*) and the success rate in accurately identifying smaller flows (*true negative rate*).

In [14], Liu et al. recommend the application of a random forest decision tree for pinpointing eight essential features crucial for developing a classification model. They introduce a dual-layered architectural framework that involves an initial pre-classification phase at the edge devices within an SDN setup, followed by a more detailed classification at the network’s central controller. This classification scheme distinguishes between four distinct types of flows: elephant, cheetah, tortoise, and porcupine, each representing different characteristics and behaviors within the network traffic. The research primarily evaluates the effectiveness of this system based on two metrics: the precision of the flow classification and the time delay associated with the classification procedure.

In their research, Hamdan et al. [15] introduce a two-level classification framework for network traffic, initially sorting flows at switches and finalizing classifications at the central controller. This method uses the count-min sketch algorithm at the switch level to separate mice from potential elephant flows, with a decision tree at the controller for final decisions. The system’s algorithms are periodically refreshed with data from the controller, emphasizing classifier accuracy, and validated with real traffic models.

He et al. [16] and Qian et al. [17], in 2022, each proposed sketch-based solutions for flow table optimization. He et al. developed a single-level, lightweight scheme, while Qian et al. introduced TCAM-based storage for elephant flow labels to balance accuracy between elephant and mouse flow identification. Both studies utilized real Internet Service Provider (ISP) packet traces for evaluation, indicating the practical effectiveness of their approaches in traffic management.

In the study [18], da Silva et al. introduced a predictive model using the Locally Weighted Regression (LWR) algorithm to estimate the size and duration of new network flows by examining patterns from previous flows and their immediate correlations. Following up, in 2022, employing a hashing mechanism inspired by the Cuckoo Search meta-heuristic for enhanced flow management [19] was proposed by the same authors. Pekar et al. presented a novel threshold-agnostic heavy-hitter classification system [20], which utilizes template matching to identify elephant flows based on the packet size distribution observed in the initial packets, offering a nuanced method for flow classification without predetermined thresholds.

The CrossBal system, detailed in [21], is a hybrid load-balancing solution that employs Deep Reinforcement Learning (DRL) to specifically address elephant flows through a three-level detection mechanism, including threshold-based filtering, followed by rerouting for efficient load distribution. In a related study, Wassie et al. [22] introduced a deep learning approach utilizing deep autoencoders, gradient boosting, and autoML predictive algorithms like eXtreme gradient boosting (XGBoost) [23] and the gradient boosting machine (GBM) [24], aimed at enhancing flow management.

All the mentioned studies focus on classifying flows after observing several initial packets. However, our goal is to identify a flow as quickly as possible, ideally based on the information carried in the first packet. Durner et al. [25] achieved flow classification using just the first packet’s 5-tuple data and its size. Hardegen et al. [26] proposed using multiclass prediction instead of binary classification (elephant/mouse) with a deep neural network to predict flow characteristics from the first packet’s 5-tuple. This approach follows a similar methodology to their earlier work [27] on predicting a flow’s bit rate from the first packet’s 5-tuple.

Regarding the most recent works, in 2023 Gomez et al. [28] evaluated several machine learning algorithms for classifying flows from the first packet. Similar to other studies, it focused on classification accuracy and not on flow table impact or traffic coverage. Xie et al.’s 2024 paper [29] proposed a two-stage decision tree system for elephant flow classification. The first stage is utilizing information contained in first packet headers. The system was developed in P4, but tested only in an emulator, lacking real-device validation.

Recent studies have also applied neural networks for network flow classification with a focus on QoS rather than traffic engineering. Alkhalidi et al. [30] introduced a one-dimensional convolutional neural network for classifying flows into various classes using packet header information. A notable innovation is the automatic selection of specific packet header bits, reducing feature count, processing time, and energy consumption while maintaining satisfactory accuracy. Yaseen et al. [31] employed a similar approach to classify traffic and assign the Differentiated Services Code Point (DSCP) field, implementing their system within an SDN controller and testing it in the Mininet emulator for emergency traffic prioritization scenarios.

As can be seen, the referenced studies focus on flow classification accuracy or true positive/true negative rates. However, they neglect the practical implications of these algorithms for traffic engineering goals. For instance, misclassifying the largest flow in a network can substantially impact traffic coverage, far more than the misclassification of smaller flows. The metrics employed in these studies do not account for such nuances. Specifically, there has been a lack of focus on metrics essential for traffic engineering, such as the reduction in flow table entries or the volume of traffic managed after classification. These aspects are critical for assessing the load on switches and controllers, as well as for understanding the broader effects on traffic engineering strategies and overall system performance. Moreover, an analysis of the significance and entropy of the information contained in the first packet’s 5-tuple—specifically, identifying which fields are crucial for detecting an elephant flow at its inception—has not yet been addressed.

## 3. Methodology

Predicting the size of a flow based on its initial packet is achievable with a type of machine learning known as regression. Regression, a principal method of supervised learning, requires labeled input data to train the model for predictive tasks. The TabNet model utilized in this research is available in the GitHub repository: https://github.com/dreamquark-ai/tabnet (accessed on 20 June 2024).

### 3.1. Training Environment

Training was conducted on a high-performance machine equipped with the following specifications:

- **Memory:** 128 GB RAM
- **Graphics Processing Unit (GPU):** NVIDIA GeForce RTX 4090 with 24 GB of VRAM
- **Central Processing Unit (CPU):** Intel Core i9-13900KF with 24 cores

These resources were more than sufficient to conduct the training and validation processes. In fact, the computational resources were not consumed beyond 20%, even when using the most demanding hyperparameter combinations. Training times for a single epoch ranged from as short as 10 s to as long as 2 min, depending on the complexity of the hyperparameter configurations. This ample capacity ensured efficient handling of the large dataset and complex computations involved in training the TabNet model, facilitating timely convergence and optimal performance.

Inference, which involves using the trained model to make predictions on a validation dataset, was also highly efficient. Inference latency varied from 10 to 23 s, depending on the model complexity and the size of the validation dataset (maximum 1,303,496; minimum 130,349). This capability is crucial for practical deployment in high-speed network environments where timely decision-making is essential.

### 3.2. Dataset

The effectiveness of an ML algorithm is significantly influenced by the dataset it is trained on. In our study, we base our evaluation on data that includes length and size distributions of flows, collected from a large campus network over 30 days [1]. For processing these data, we employed the package described in [32].

The dataset in question comprises over 4 billion flows, with its complete flow records amounting to approximately 278 GB in binary format. Given this immense size, we used an anonymized subset of the data for training and evaluating our models, as published in [33]. This subset represents one hour of traffic, encompassing 6,517,484 flows and 547 GB of data transmission. This specific time frame was chosen to ensure it was free of anomalies and that the theoretical reduction rate curve of a perfect elephant classifier for this hour closely mirrors that of the complete 30-day dataset. In the published open-source dataset, IP addresses were anonymized using the prefix-preserving Crypto-PAn algorithm [34]. As demonstrated in [33], this anonymization process does not affect the performance of the ML models.

### 3.3. Input Features

The input data, sourced from the flow 5-tuple, encompasses the source IP address, destination IP address, transport layer source port, transport layer destination port, and transport layer protocol, cumulatively contributing to 104 bits. Our investigation focuses on two distinct representations of this input data:

- **Bits**: Each header field is segmented into separate bits, yielding 104 unique features, which are denoted as binary values (0 or 1).
- **Octets**: Headers that exceed 8 bits in length, such as IP addresses or ports, are segmented into distinct octets. This approach produces 13 features, with each feature represented as an 8-bit integer.

### 3.4. Balancing the Dataset

Achieving a balanced training dataset was key to the effectiveness of the model. In our initial training dataset of 5,213,988 flows, mouse flows greatly outnumbered elephant flows, necessitating measures to balance this disparity for optimal accuracy. The results discussed in this paper stem from the model trained on a balanced dataset, achieved through various ratios, following these steps:

1. Define the *ratio*, e.g., 10%.
2. Calculate the *balanced dataset size* as the size of the initial training dataset multiplied by the ratio (5,213,988 × 10% = 521,398 flows).
3. Organize the initial training dataset in descending order, with the largest flows positioned at the start.
4. Extract the top half of the *balanced dataset size* number of flows from the start of this sorted list.
5. Randomly select the remaining half of the *balanced dataset size* number of flows from the rest of the initial dataset.

### 3.5. Training

**Figure 1:**
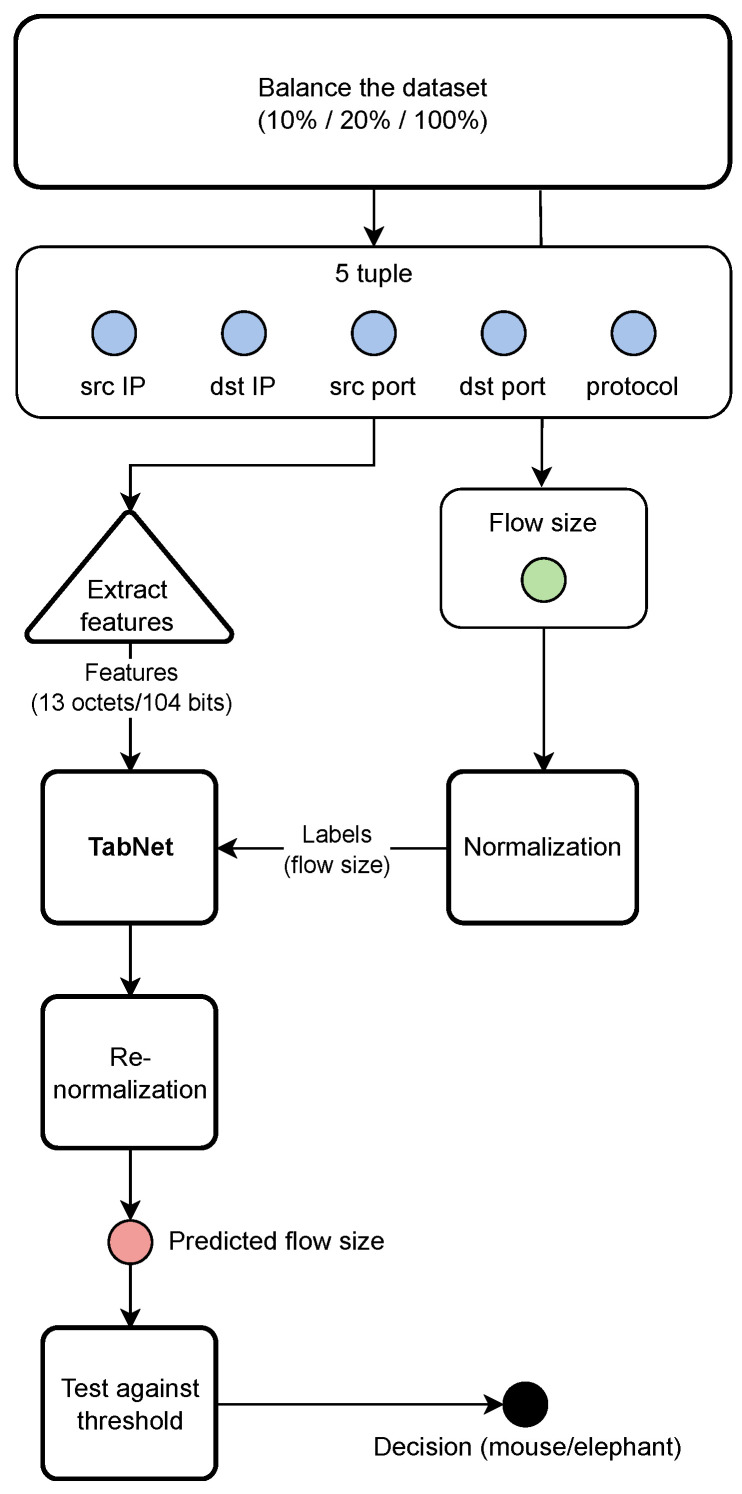
Training and evaluation of the TabNet model.

This phase encompasses the selection of hyperparameters, normalization of labels, and the model training process. The workflow of the training phase is depicted in Figure 1.

The model underwent training on a shuffled, balanced training dataset before its performance was assessed using the validation dataset. Training and validation were carried out with several combinations of hyperparameters. The hyperparameters that were varied are listed in Table 1, while those that remained unchanged throughout all training sessions are detailed in Table 2. We also present the table with the parameters that varied and are coupled directly to the TabNet model in Table 3.

The batch size was varied to observe its effect on model convergence and generalization. The selected range of 2560 to 10,240 was chosen based on several considerations:

- **Computational Efficiency:** Batch sizes in the range of 2560 to 10,240 were selected to balance between memory usage and computational efficiency. Very small batch sizes might lead to inefficient Graphics Processing Unit (GPU) utilization, while very large batch sizes could exceed the memory limits of the hardware, leading to slower training times due to increased paging or the need to reduce model complexity.
- **Empirical Performance:** Preliminary experiments indicated that this range of batch sizes yielded good performance across various metrics. A batch size of 2560 provided a good trade-off between frequent weight updates and manageable noise in gradient estimates. Increasing the batch size to 5120 and 10,240 allowed for more stable training with slightly slower convergence, which was beneficial in achieving better generalization on the validation set.
- **Model and Data Characteristics:** The nature of the dataset and the model architecture also influenced the choice of batch size. Given the large dataset (5,213,988 flows) and the complexity of the TabNet architecture, batch sizes within this range were found to be effective in leveraging the computational capabilities of modern GPUs while ensuring efficient training dynamics.

Different learning rates were tested to find the optimal balance between convergence speed and stability. A lower learning rate (1 $\times \;10^{-3}$) allows for finer weight adjustments, potentially reducing the risk of overshooting minima. Higher learning rates (6 $\times \;10^{-3}$) can accelerate convergence but may require careful tuning to avoid instability. In general, a larger batch size can lead to more stable training by decreasing the likelihood of overfitting the model. In tandem with increasing the batch size, we also scaled the learning rate. This approach enabled the model to more quickly locate local minima and maxima without necessitating a proportional adjustment in the number of epochs. The use of different loss functions, MAE and MSE, allows us to assess their impact on regression performance. MAE is less sensitive to outliers compared to MSE, which penalizes larger errors more heavily. Specific balancing dataset ratios were chosen to ensure a sufficient number of elephant flows were included without overwhelming computational resources. A 10% ratio provides a conservative balance, while a 20% ratio allows for a more comprehensive inclusion of elephant flows. The 100% ratio indicates no rebalancing was performed, serving as a control to compare against the balanced scenarios. These steps ensure that minority classes are adequately represented, improving the model’s ability to generalize across different traffic types while keeping the computational expense manageable.

The model was trained for up to 200 epochs to ensure sufficient learning time for convergence. This duration was selected based on preliminary experiments indicating that 200 epochs allow the model to adequately learn from the data without overfitting. However, training did not always take the full 200 epochs thanks to the early stopping feature, which halted training when no significant improvement in performance was observed over a set number of epochs. The Adam optimizer was chosen for its adaptive learning rate capabilities, which can improve convergence speed and stability.

Varying the width of this layer (8, 16, 32) allows us to investigate the impact of model capacity on performance. A wider layer can capture more complex patterns but may also increase the risk of overfitting. Similar to the decision layer, varying the width of the attention embedding (8, 16, 32) helps us understand how the model’s attention mechanism scales with complexity. Wider embeddings can capture more detailed feature interactions. The number of steps (3, 6, 9) determines how many sequential decision and attention layers the data pass through. More steps can improve model performance by allowing more complex transformations but at the cost of increased computational requirements.

Training and validation were conducted with various normalization techniques. We explored two distinct approaches to **label normalization**, designated as NONE, and MINMAX:

- **NONE** refers to the absence of label normalization. Models are trained and assessed using the unaltered labels, which vary from 64 bytes (minimum flow size) to 3,218,210,994 bytes (maximum flow size).
- **MINMAX** is a transformation where its minimum value becomes 0, its maximum value becomes 1, and all other values are scaled proportionally to fall within the range of 0 to 1. The procedure is detailed in Equation (1).

Let $\mathrm{labels}=\{l_{1},l_{2},\ldots ,l_{n}\}$, then for each label $l_{i}$ in labels, the normalized value $T(l_{i})$ is defined by:

(1) $$T\left(l_{i}\right)=\frac{l_{i}-l_{min}}{l_{max}-l_{min}}$$

### 3.6. Model

The TabNet [7] model is a type of neural network architecture designed specifically for tabular data. Developed by researchers at Google Cloud AI, TabNet uses sequential attention mechanisms to selectively choose which features to process at each decision step, effectively enabling the model to make decisions based on important, learned features from the data. This selective feature processing allows TabNet to interpret and learn from the data in a way similar to how decision trees isolate important features but with the added flexibility and power of a neural network. TabNet’s design also promotes interpretable decision-making, which is a valuable attribute for applications requiring transparency in how input features affect predictions. This model has been shown to perform competitively on various benchmark datasets, outperforming traditional ensemble models like random forests and gradient-boosting machines in some cases.

### 3.7. Model Decision

In regression analysis, the algorithm predicts a continuous outcome, which, for our study, corresponds to the anticipated flow size in bytes. To illustrate the relationship between flow table reduction and traffic coverage, retraining and refitting the model repeatedly is unnecessary. We can simulate decision-making adjustments by modifying the threshold for classifying a flow as an elephant based on its predicted size. Here, the term *label* denotes the true flow size as extracted from the dataset.

### 3.8. Evaluation

Current research in the field largely neglects metrics essential for assessing the effectiveness of flow-based traffic engineering. Many studies emphasize the accuracy of flow classification, measuring success through parameters such as the true positive rate, true negative rate, and precision in predicting flow size and duration. Yet, these metrics offer limited insights into the practical implementation of algorithms in this research area. Crucially, the misclassification of a network’s largest flow disproportionately affects overall traffic coverage compared to the misclassification of smaller flows. **The metrics commonly used in existing literature fail to capture this significant disparity. Apart from the metrics, it is also unknown which information is the most important for predicting which flow belongs to which class**.

To bridge these gaps, we introduce new metrics specifically designed to evaluate ML models in the context of detecting elephant flows for traffic engineering purposes. We employ two particular metrics for this evaluation: **the reduction in the number of flow table entries created** and **the percentage of traffic covered**. These metrics are intended to provide a more relevant assessment of how well the models perform in practical traffic management scenarios, focusing on optimizing network efficiency and capacity utilization.

It is important to understand the inherent trade-off between these metrics. Increasing the threshold for elephant flow detection results in a larger reduction in the number of flow table entries, but it also diminishes the percentage of traffic that is covered. Striking the right balance between these factors is crucial for optimizing network efficiency and maintaining high QoS.

Additionally, we assess the **information contained in the input 5-tuple (entropy) and its significance in influencing the output predictions’ (feature importance)**. Our analysis explores the impact of information across the two proposed input data approaches. This study aims to provide a more in-depth understanding of which elements of the input data are more relevant than others.

## 4. Results

Out of 504 distinct results (two input data types, three dataset ratios, seven TabNet hyperparameter combinations, three batch size and learning rate combinations, two loss functions, and two normalization types) we selected the best result per input data type and dataset ratio. In this research, the best means the largest flow table reduction at 80% traffic coverage.

### 4.1. Flow Table Reduction vs. Traffic Coverage

The visual representations illustrate the reduction in flow table operations and achieved traffic coverage. Remarkably, the y-axis exhibits a logarithmic scale. On the y-axis, each unit corresponds to a multiplier (e.g., 1000 indicates a reduction by a factor of 1000, resulting in the number of created flow table entries being 1/1000 of its original value). The goal is to minimize creation of individual flow entries while preserving optimal traffic coverage. A model is deemed more effective as its curve approaches the top-right corner of the graph.

The black line, identified as **Data**, illustrates the projected performance derived from the validation dataset, comprising 1,303,496 flows. This projection is predicated on the assumption of perfect prediction of each flow’s size on its initial packet. This methodology, described in [35] as the *first* method, involves selecting the smallest subset of the largest flows, arranged by size in descending order, which collectively represent a predetermined percentage of the total network traffic.

**Figure 2:**
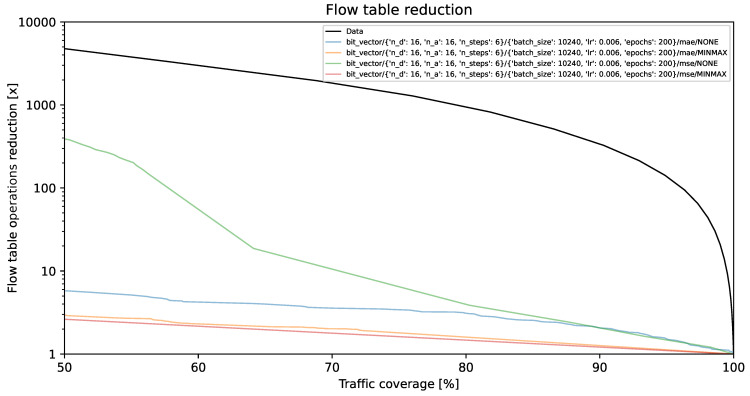
Flow table reduction for the balanced dataset with *10% ratio* and *bit vector* input data.

**Figure 3:**
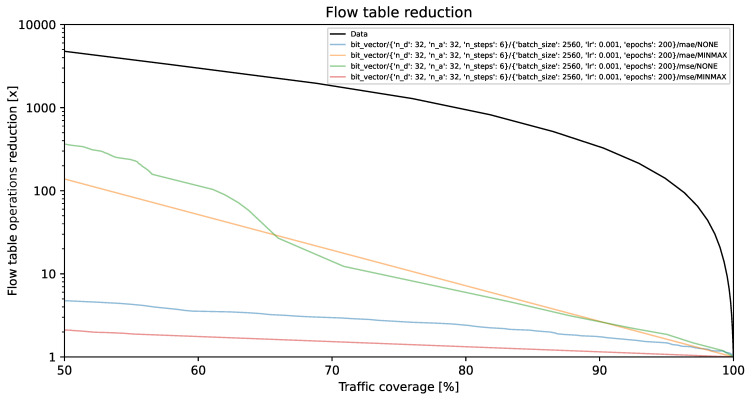
Flow table reduction for the balanced dataset with *20% ratio* and *bit vector* input data.

**Figure 4:**
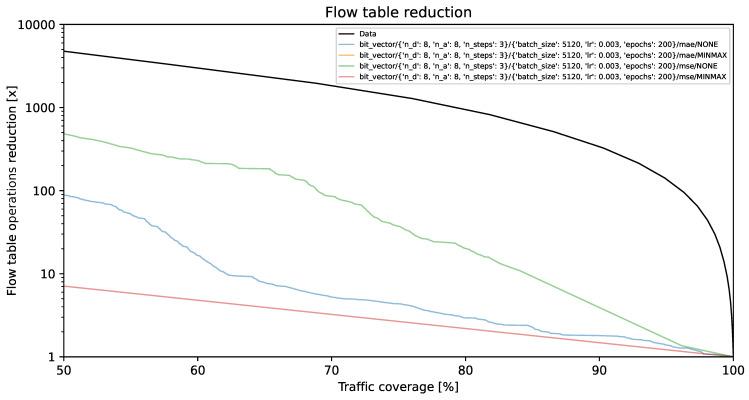
Flow table reduction for the unbalanced dataset (*100% ratio*) and *bit vector* input data.

**Figure 5:**
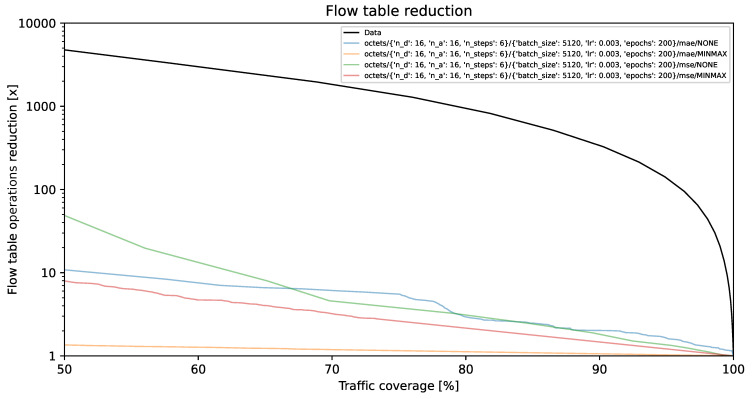
Flow table reduction for the balanced dataset with *10% ratio* and *octets* input data.

**Figure 6:**
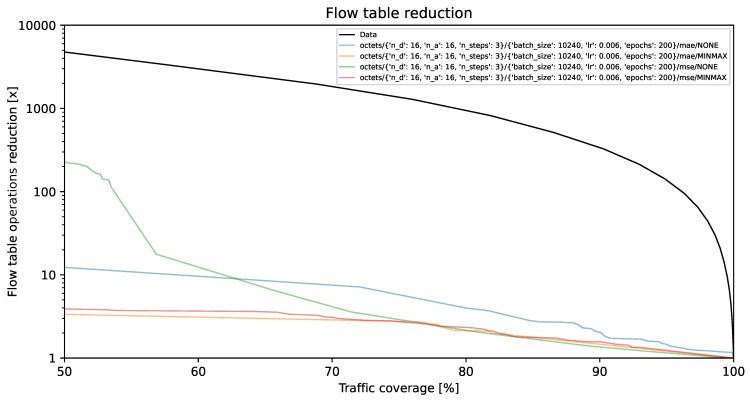
Flow table reduction for the balanced dataset with *20% ratio* and *octets* input data.

**Figure 7:**
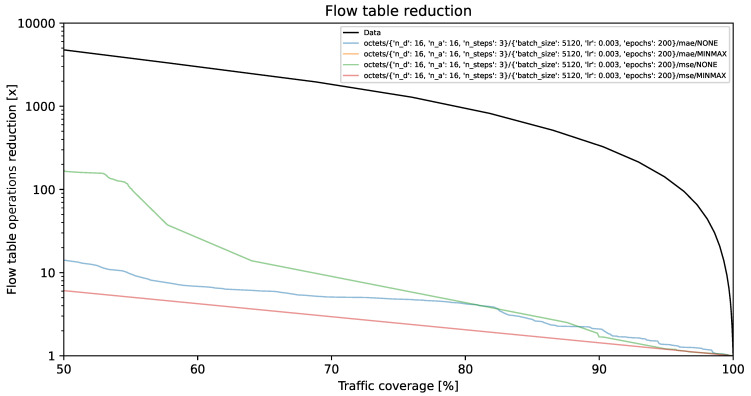
Flow table reduction for the unbalanced dataset (*100% ratio*) and *octets* input data.

Figure 2, Figure 3 and Figure 4 present results for bit vector input data representation and the balanced dataset with ratios of 10%, 20%, and 100%, whereas Figure 5, Figure 6 and Figure 7 present results for octet input data representation and the balanced dataset with ratios of 10%, 20%, and 100%. Additionally, as seen in Figure 4 and Figure 7 we were unable to draw the reduction vs. coverage result for the MAE with MINMAX normalization type, due to the fact that obtained results did not fit in the traffic coverage area of interest (50–100%). It seems that the model in these configurations was extremely underfitted, and it was not able to sufficiently recognize trends and patterns based on the input data.

In Table 4, we presented the five top-performing configurations. The table illustrates how varying training configurations can impact the effectiveness of TabNet models in reducing created flow entries number while maintaining constant 80% traffic coverage.

### 4.2. Feature Entropy and Importance

**Figure 8:**
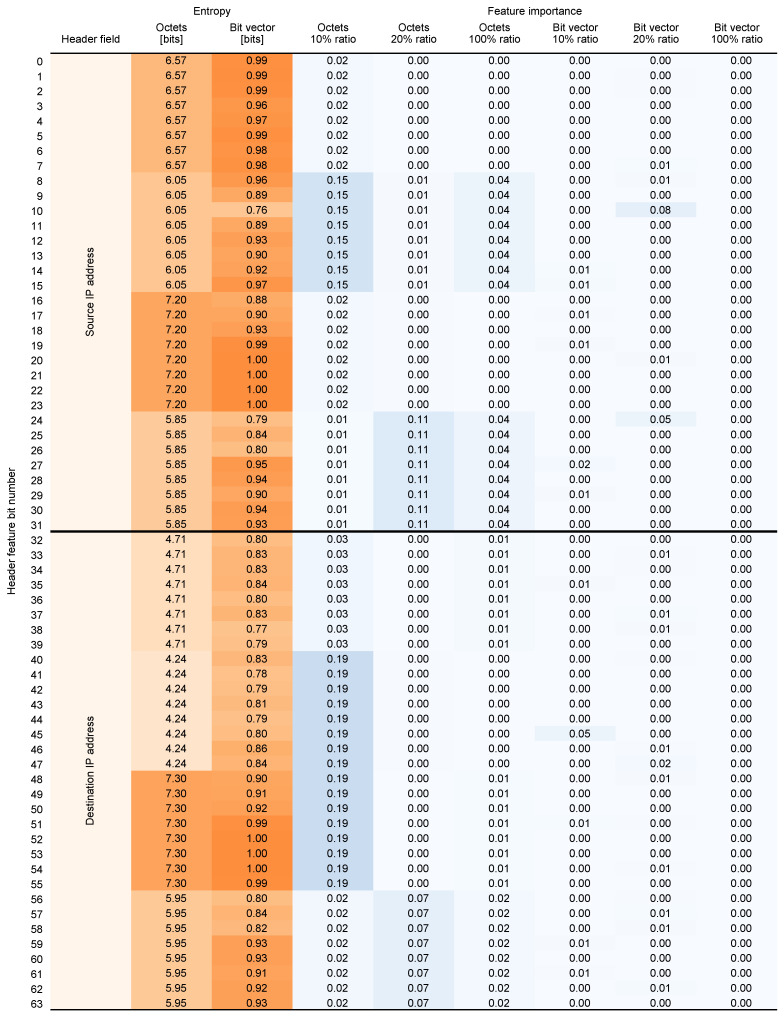
Feature entropy (calculated) and importance (from TabNet) (bits 0–63).

**Figure 9:**
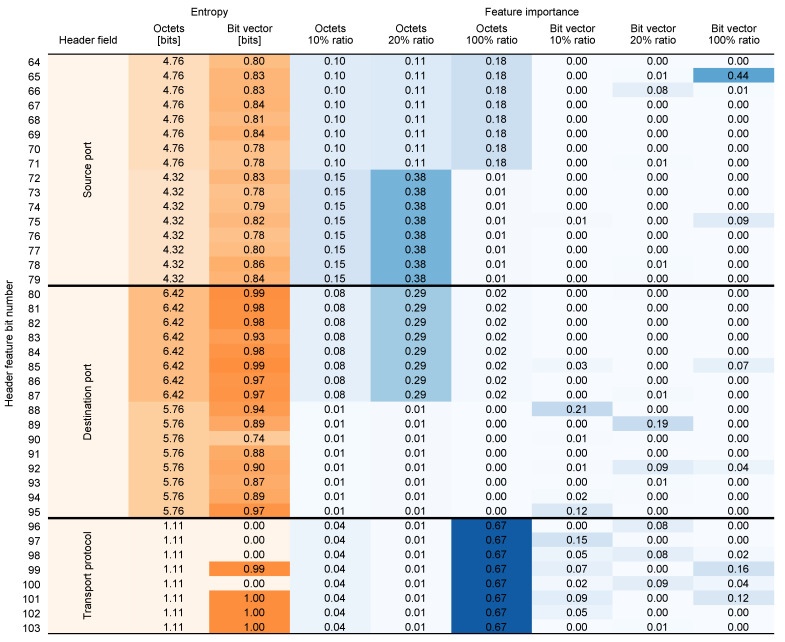
Feature entropy (calculated) and importance (from TabNet) (bits 64–103).

To provide additional insight into which features are most essential for providing an accurate prediction, we performed an analysis of the information amount contained in the input 5-tuple (feature entropy) and its significance in influencing the model (feature importance). The results of the analysis are presented in Figure 8 and Figure 9.

Feature importance analysis was performed for the all input data variations and all dataset balancing ratios. In entropy analysis, we calculated the entropy for both input data representations. In this context, entropy measures the average amount of information contained in a feature (byte or bit, depending on the input data representation). Higher entropy indicates greater randomness, while lower entropy indicates less varied values. We express the entropy in bits. This tells us how many bits on average are needed to encode the information contained in a particular feature.

## 5. Discussion

**Figure 10:**
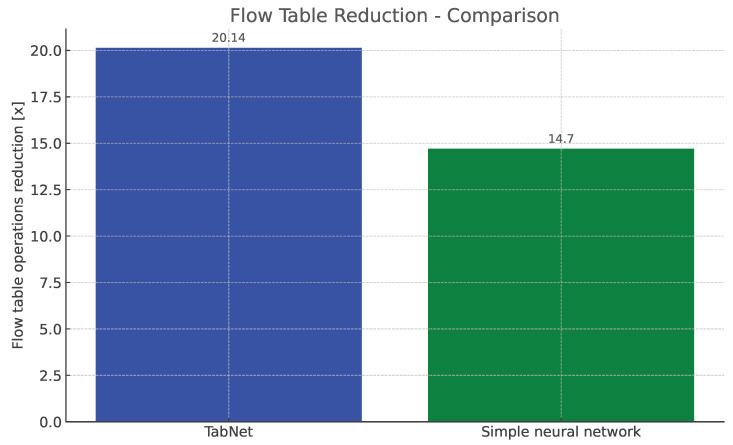
Comparison of TabNet and the best neural network from [36].

The flow table reduction results show the superiority of MSE over the MAE loss functions. MSE employs an error amplification mechanism. For larger errors, the squared term magnifies their impact, which accelerates the minimization process during training. MSE amplifies the influence of outliers, which seems to fit our scenario much better than the MAE, which, on the other hand, treats all errors equally, minimizing the impact of outliers on the loss function. Additionally, as can be seen in the reduction results, TabNet worked much better on unnormalized labels rather than normalized labels. Regarding the TabNet parameters, the best results were obtained with the width (both the decision prediction layer and attention embedding for each mask) set to 8. The best reduction rate achieved for the 80% traffic coverage was 20.14. As shown in Figure 10 this is a 25% higher reduction rate than achieved previously with neural networks comprising solely linear layers, which provided only 15-fold reduction for the best parameter combination [36].

Feature entropy analysis shows that the most predictable fields are related to the transport protocol, and source port in both input data representations. This is expected, as the transport protocol field contains mostly one of the two values: 6 for TCP and 17 for UDP. The least predictable (most random) fields are the addresses (both source and destination) and destination port.

As the results show, only a fraction of initial input data is significant for the model in predicting the flow size. Features are also not equally important across dataset balancing ratios. In the *octets* data, different features like transport protocol, ports, and addresses dominate depending on the ratio. For the 100% ratio, the transport protocol has significantly higher weight than the other features. For the 20% ratio, the source and destination ports are the most important, while for the 10% ratio, the source and destination addresses are the most important. Conversely, in the *bit vector* data, the transport protocol and destination port are consistently important, while the source and destination addresses are not, and the source port’s importance declines at lower ratios.

The variation in feature importance across different dataset ratios can be attributed to the nature of the balancing process itself. At higher ratios, where the dataset is more imbalanced, the model may rely heavily on more generalized features such as transport protocols that are universally present in all flows. However, at lower ratios, where the dataset is more balanced, the model can discern more nuanced patterns and dependencies, leading to a higher significance of specific features like source and destination addresses. This deeper exploration reveals that feature importance is inherently tied to the composition and characteristics of the training data, impacting the model’s predictive behavior depending on the dataset’s balance.

## 6. Conclusions

As demonstrated in this study, employing a TabNet model to identify elephant flows from the initial packets enables a reduction in the number of flow table entries by approximately 20-fold while still encompassing 80% of the traffic. The reduction in number of required flow table entries can not only enable flow-based traffic engineering on switches with limited capacities but also positively influence flow table lookup, consequently enhancing the switching rate. We also evaluated the significance of the information carried by the initial packet 5-tuple. It was determined that only a subset of all features is truly important for the model in providing accurate results. Utilizing this subset of the input data, one can achieve faster training and inference time, which can result in quicker elephant flow classification and minimization of the additional latency.

## Figures and Tables

**Table 1:** Variable hyperparameters.

Batch Size	Learning Rate	Loss Function	Balancing Ratio
2560	1 $\times \;10^{-3}$	Mean Absolute Error (MAE)	10%
5120	3 $\times \;10^{-3}$	Mean Square Error (MSE)	20%
10,240	6 $\times \;10^{-3}$		100% (no balancing)

**Table 2:** Constant hyperparameters.

Epochs	Optimizer
200	Adam

**Table 3:** TabNet parameters.

Width of the Decision Prediction Layer	Width of the Attention Embedding for Each Mask	Number of Steps in the Architecture
8	8	3
16	16	6
32	32	9

**Table 4:** Top 5 results and hyperparameter values for the 80% traffic coverage.

Input	Dataset Ratio	Model Parameters	Normalization	Loss Function	Batch Size	Learning Rate	Flow Table Reduction (Best)	Flow Table Reduction (SD)
bits	100%	8/8/3	NONE	MSE	5120	0.003	20.14	5.32
bits	100%	16/16/3	NONE	MSE	2560	0.001	18.74	3.79
bits	100%	8/8/3	NONE	MSE	2560	0.001	14.25	2.98
bits	10%	32/32/3	NONE	MSE	10,240	0.006	12.92	2.61
bits	20%	16/16/3	NONE	MSE	2560	0.001	12.59	2.51

## Data Availability

The anonymized input data are available in the GitHub repository: https://github.com/piotrjurkiewicz/flow-models (accessed on 20 June 2024).
